# Deubiquitinating Enzymes: A Critical Regulator of Mitosis

**DOI:** 10.3390/ijms20235997

**Published:** 2019-11-28

**Authors:** Jinyoung Park, Jinhong Cho, Eunice EunKyeong Kim, Eun Joo Song

**Affiliations:** 1 Molecular Recognition Research Center, Korea Institute of Science and Technology, Seoul 02792, Korea; jypark@kist.re.kr; 2 Biomedical Research Institute, Korea Institute of Science and Technology, Seoul 02792, Korea; wlsghd1116@gmail.com (J.C.); eunice@kist.re.kr (E.E.K.); 3 Graduate School of Pharmaceutical Sciences and College of Pharmacy, Ewha Womans University, Seoul 03760, Korea

**Keywords:** mitosis, ubiquitination, deubiquitination, cancer

## Abstract

Mitosis is a complex and dynamic process that is tightly regulated by a large number of mitotic proteins. Dysregulation of these proteins can generate daughter cells that exhibit genomic instability and aneuploidy, and such cells can transform into tumorigenic cells. Thus, it is important for faithful mitotic progression to regulate mitotic proteins at specific locations in the cells at a given time in each phase of mitosis. Ubiquitin-dependent modifications play critical roles in this process by regulating the degradation, translocation, or signal transduction of mitotic proteins. Here, we review how ubiquitination and deubiquitination regulate the progression of mitosis. In addition, we summarize the substrates and roles of some deubiquitinating enzymes (DUBs) crucial for mitosis and describe how they contribute error correction during mitosis and control the transition between the mitotic phases.

## 1. Introduction

In general, a tumor is caused by abnormal cells that undergo unrestricted divisions and proliferation. These events are the same as those that manifest upon the failure to control mitosis, and such a failure eventually results in cell death or tumorigenesis. Mitotic defects can occur at any phase of mitosis. Specifically, dysregulation of the spindle assembly checkpoint (SAC) leads to prolonged mitotic arrest and constitutes the major cause of several mitotic defects. Hence, the SAC is an important target for the development of antiproliferative chemotherapeutic strategies [1]. Moreover, several cellular components, including microtubules, mitotic kinases, motor proteins, and various multiprotein complexes, have been targeted for mitosis-based cancer therapies [2]. These cellular components are regulated via post-translational modifications (PTMs), such as phosphorylation, acetylation, glycosylation, ubiquitination, and deubiquitination. In this review, we will focus on mitosis-related ubiquitination and deubiquitination processes, and the substrates at each phase associated with them.

### 1.1. Mitosis

The cell cycle refers to a series of processes including DNA synthesis (S phase), cell growth (G1 phase), evaluation of the accuracy of the genomic materials (G2 phase), and cell division (M phase). Of these phases, mitosis (M phase), which occurs only in eukaryotic cells, is an important step in ensuring the stability of the entire genome by duplicating the genetic information and equally segregating it into two daughter cells [3]. Mitosis can be divided into six stages, including cytokinesis.

(1) Prophase: Mitosis begins with the nuclear envelope breakdown (NEBD), an essential step for spindle assembly, followed by condensation of replicated DNA in the chromosome. During prophase, the two duplicated centrioles move to the opposite poles, where each pair forms a centrosome. The two centrosomes then nucleate the polymerization of microtubules from the opposite ends, forming the spindle.
(2) Prometaphase: This phase is a dynamic part of mitotic progression. Microtubules rapidly assemble and disassemble by growing out from the duplicated centrosomes to find the accurate attachment site at the kinetochores of the chromosomes. The attached microtubules pull each chromosome from the opposite sites until all the chromosomes are bi-oriented and aligned.
(3) Metaphase: The assembly of the mitotic spindle and its correct attachment to the kinetochore of sister chromatids are stabilized, completing the alignment of sister chromatids at the equator of the spindle for proper segregation of chromosomes toward the opposite poles of the spindle [3,4,5]. However, kinetochore–microtubule attachment is prone to errors, and any such error may result in chromosome misalignment. The SAC is a complex network of regulatory factors involved in the resolution of such errors [6]. It delays the chromosome segregation until all chromosomes are correctly attached to the spindle apparatus at their kinetochores and all kinetochores have sufficient occupancy and tension by the spindle microtubules. Thus, the SAC is a quality control mechanism involved in the maintenance of genomic stability [7,8].
(4) Anaphase: After the requirements of the SAC are satisfied, the cell enters anaphase. During this phase, microtubules attached to the duplicated chromosomes shorten from the opposite sites, separating the chromosome pairs and pulling each chromosome of a pair toward opposite spindle poles [5]. Following successful chromosome segregation, the spindle microtubules undergo a dramatic reorganization, forming the spindle mid-zone [9].
(5) Telophase: Once all the chromosomes reach their poles, the final phase of mitosis, termed telophase, begins. During telophase, the nuclear envelope reforms around the nuclei of daughter cells and chromosomes decondense [5].
(6) Cytokinesis: This step refers to the division of the cytoplasm into two daughter cells. A cytokinetic furrow formed by the contraction of the actomyosin ring splits the cytoplasm into two domains. At this stage, two daughter cells remain connected by a narrow intracellular bridge containing antiparallel bundles of microtubules that overlap at the mid-body. The physical separation of the two daughter cells is finally accomplished by the fission of the plasma membrane via the process called abscission [3,10].

### 1.2. Ubiquitination and Deubiquitination

Ubiquitination is a reversible PTM, which involves the covalent attachment of the small conserved protein ubiquitin (Ub) to a target protein, almost exclusively at a lysine residue [11]. It requires the concerted interplay of three different enzymes [12]. E1 Ub-activating enzymes bind to both ATP and ubiquitin and expose the ubiquitin’s active site containing a cysteine residue, with the release of an AMP. E2 Ub-conjugating enzymes take over activated ubiquitin from E1 enzymes and cooperate with E3 Ub ligase. The E3 enzymes interact with E2 enzymes and recruit specific substrates to promote conjugation of single ubiquitin or polymeric ubiquitin chains [13]. Ubiquitination has been known to regulate proteasomal and lysosomal protein degradation, protein–protein interactions, protein localization, and activation of cellular signaling pathways [14].

Deubiquitination is the reverse process of ubiquitination, that is, excise the conjugated monoubiquitin or polyubiquitin chains from the modified proteins [4]. This process is catalyzed by a large group of proteases called deubiquitinating enzymes (DUBs). Their physiological roles include controlling protein stability and quality, maintaining ubiquitin homeostasis, and regulating ubiquitin signals against the functions of E3 Ub ligase. Therefore, DUBs can regulate numerous cellular events such as cell cycle, DNA damage response, inflammatory signaling, and cell death [11]. The fact that the human genome encodes approximately 100 DUBs is a testament to their broad-spectrum involvement in cellular events [11,15].

Although DUB paralogs are highly conserved in function, they can be divided into the following six families based on the architecture of their catalytic domains: ubiquitin-specific proteases (USPs), ubiquitin C-terminal hydrolases (UCHs), ovarian tumor proteases (OTUs), Machado-Josephin proteases (MJDs), JAB1/MPN/MOV34 (JAMMs), and MIU-containing novel DUB family (MINDY). The first four families are cysteine proteases, whereas JAMMs are metalloprotease [15]. MINDY is the most recently discovered DUB family by Rehman and colleagues. This DUB family has a catalytic domain that is a new folding variant within the cysteine protease superfamily and shows a remarkable selectivity for cleaving long lysine 48 (Lys48)-linked ubiquitin chains. In particular, cleavage selectivity of DUBs is determined by catalytic domain alone, whereas MINDY requires motif interacting with ubiquitin (MIU) as well as catalytic domain for maximal DUB activity [16]. The enzymatic activities of cysteine protease DUBs depend on the catalytic triad composed of three crucial amino acid residues, including cysteine (Cys), histidine (His), and aspartic acid (Asp) or asparagine (Asn). The presence of a His residue adjacent to the Cys residue lowers the pKa of the Cys residue, facilitating a nucleophilic attack, where the His residue is aligned and polarized by the third residue (Asp or Asn) [17]. All cysteine protease DUBs are covalently linked to the C-terminus of the distal ubiquitin to form acyl intermediates [18]. In distinction to cysteine protease DUBs, JAMMs generally coordinate zinc ions with His, Asp, and serine (Ser) residues, which activate a water molecule to attack the isopeptide bond between the ubiquitin and substrate [11].

## 2. E3 ubiquitin Ligases Involved in Mitosis

Several E3 ligases participate in mitotic control at almost every phase. Of these, the anaphase-promoting complex/cyclosome (APC/C) E3 ligase plays an important part, as it controls the metaphase-to-anaphase transition by ensuring accurate chromosome segregation and regulates the mitotic exit by mediating the degradation of key mitotic cyclins [19,20,21]. APC/C is activated by binding to one of its co-activators, CDC20 or CDH1. CDC20 associates with the phosphorylated APC/C during early mitosis and leads to the ubiquitination and degradation of securin and cyclin B1 after all chromosomes are bi-oriented on the mitotic spindle and the SAC is silenced, inducing the onset of anaphase [22,23,24,25,26,27,28,29]. However, during late mitosis and throughout G1, APC/C binds to dephosphorylated CDH1 [30]. The resulting APC/C–CDH1 complex ubiquitinates and degrades CDC20 and late mitotic kinases, such as Aurora kinases (Aurora A and B) and polo-like kinase 1 (PLK1), thereby promoting the mitosis exit [26,31].

Modulation of the localization of Aurora B affects its functions during mitosis. During metaphase, Aurora B participates in the destabilization of erroneous microtubule attachments at the kinetochores [32,33]. During anaphase, it accumulates at the spindle mid-zone, and this accumulation is required to initiate cytokinesis [34]. Non-proteolytic ubiquitination of Aurora B by Cullin3-RING ubiquitin ligases (CRL3)-based complex (CRL3-BTB domain (protein–protein interaction motif)-containing adaptor complex) regulates the translocation of Aurora B during the transition from metaphase to anaphase. CRL3-KLHL9/13-dependent polyubiquitination dissociates Aurora B from mitotic chromosomes, and CRL3-KLHL21-dependent monoubiquitination promotes its subsequent translocation to the spindle mid-zone [35,36]. CRL3-KLHL22-mediated ubiquitination of PLK1 triggers its rapid dissociation from the kinetochores [37]. In fact, PLK1 stabilizes the accurate kinetochore–microtubule attachments, but it should be released from the kinetochores upon bi-orientation of the chromosomes [38]. Overall, E3 ligases involved in mitosis operate to trigger timely chromosome segregation and ensure genomic integrity [29].

## 3. Deubiquitinating Enzymes Involved in Mitosis

**Figure 1:**
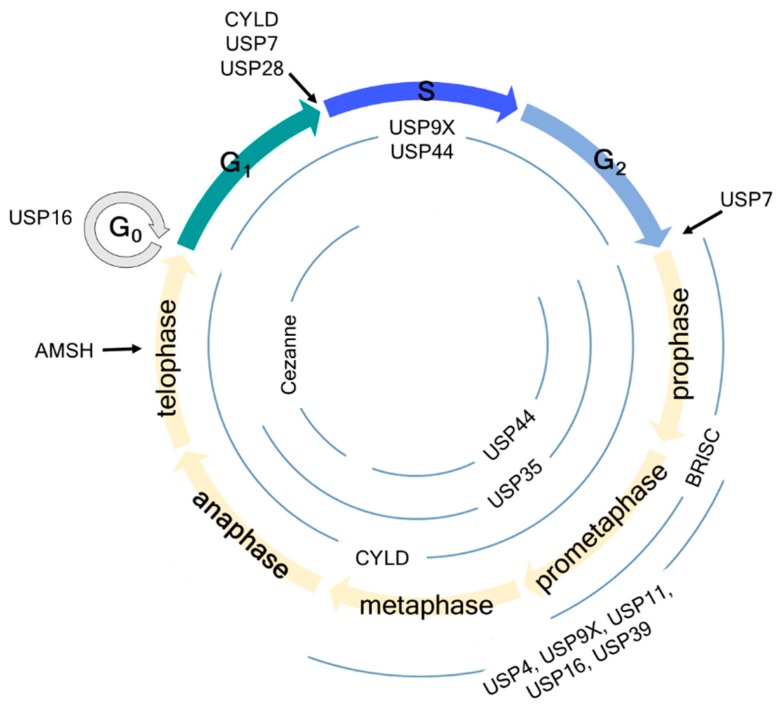
Schematic overview of cell cycle regulation by deubiquitinating enzymes (DUBs). USP7 regulates the G1-S and G2-M checkpoints by deubiquitinating claspin. USP16 regulates the G0 and early M phases by deubiquitinating histone H2A and PLK1, thereby regulating chromosome segregation and alignment. USP28 regulates the G1-S checkpoint by deubiquitinating p53 independent of the SAC. AMSH regulates the late M phase by deubiquitinating vesicle-associated membrane protein 8 (VAMP8). Information about USP44, USP9X, Cylindromatosis (CYLD), USP35, and Cezanne is detailed in the manuscript.

**Figure 2:**
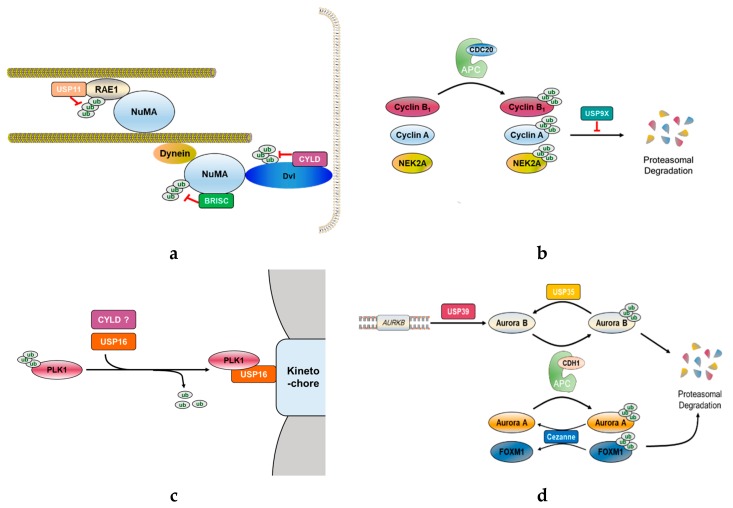
Functional role of mitosis-related DUBs. (**a**) CYLD deubiquitinates Disheveled (Dvl), which stabilizes astral microtubules by forming Dvl-NuMA-dynein/dynactin complex. Another DUB, BRISC, adjusts spindle assembly by deubiquitinating NuMA and negatively controlling its association with dynein and importin-β. Furthermore, USP11 also regulates proper mitotic spindle formation. Microtubules interact with NuMA through RAE1, where USP11 deubiquitinates RAE1 to coordinate its functional interaction with NuMA. (**b**) In early mitosis, some mitotic proteins, such as cyclin B1, cyclin A, and NIMA-related kinase 2A (NEK2A), should be degraded for continuing mitosis. Therefore, APC/C^CDC20^ ubiquitinates and promotes proteasomal degradation of these proteins. In contrast to APC/C^CDC20^, USP9X antagonizes the proteasomal degradation of APC/C^CDC20^ substrates. (**c**) PLK1 is a potential substrate of CYLD and USP16. USP16 promotes proper chromosome alignment in early mitosis by deubiquitinating PLK1 to retain it on the kinetochores. (**d**) USP39 is involved in the splicing of Aurora B mRNA. USP35 deubiquitinates Aurora B kinase, thereby maintaining the stability of Aurora B during mitosis. To proceed with the cell cycle, APC/C^CDH1^ substrates, such as Aurora kinases and forkhead box protein M1 (FOXM1), should be degraded. Cezanne antagonizes APC/C^CDH1^ activity by deubiquitinating Aurora A and FOXM1, but it does not affect Aurora B levels.

Several DUBs have been reported involved in mitotic progression (Figure 1). For example, BRCC36 isopeptidase complex (BRISC), which specifically hydrolyzes Lys63-linked polyubiquitin chains [39,40], deubiquitinates the spindle assembly factor NuMA and negatively regulates the interaction of NuMA with dynein and importin-β, thereby indirectly regulating the function of NuMA in spindle assembly [41]. USP11, like BRISC, also regulates the functions of NuMA. It controls the ubiquitination of ribonucleic acid export 1 (RAE1), which is an mRNA export factor and known as a mitotic checkpoint regulator, at the mitotic spindle and modulates its functional interaction with NuMA, thereby indirectly regulating bipolar spindle assembly [42] (Figure 2a). Thus, different DUBs targeting different proteins may still be involved in the same mitotic event. In addition, some DUBs target multiple proteins that function at distinct mitotic phases, thus affecting various mitotic events.

### 3.1. USP44

The SAC plays an important role against chromosomal missegregation and generation of aneuploidy progeny, which is a remarkable common characteristic of tumorigenesis, by delaying sister chromatid separation until all chromosomes achieve bipolar kinetochore–microtubule attachment. Once the last chromosome bi-orients on the mitotic spindle, APC/C activated by the binding of CDC20 ubiquitinates securin and cyclin B1 to induce their proteasomal degradation and then initiates chromosome segregation [19].

USP44 is a critical regulator of the SAC. Stegmeier et al. identified that USP44 is required for efficient SAC signaling and anaphase onset. They observed that the depletion of USP44 abolishes the checkpoint function of the SAC, but overexpression of a siRNA-resistant USP44 rescues the function of the SAC in USP44-depleted cells [43]. In addition, USP44 levels and activity are increased in mitotic cells arrested in metaphase by the SAC. As the cells exit from mitosis, USP44 is rapidly degraded. Mitotic arrest-deficient 2 (MAD2) is one of the primary components of the SAC, binds to CDC20 at the kinetochores that remain unattached to microtubules and inhibits the activation of APC/C. Subsequently, MDM2-CDC20 binds to other checkpoint components, BUBR1 and BUB3, resulting in the formation of the mitotic checkpoint complex (MCC; MDM2–CDC20–BUBR1–BUB3) [44,45,46,47,48,49,50,51]. UbcH10 is an APC-specific E2 enzyme, and polyubiquitination of CDC20 by UbcH10 leads to the dissociation of MCC and activation of APC/C [52]. USP44 is thought to antagonize this process by deubiquitinating CDC20, thereby stabilizing the association between MAD2 and CDC20 and maintaining the function of the SAC [43]. Another study has suggested a novel USP44 target that regulates chromosome segregation in a SAC-independent manner. Zhang et al. showed an increased frequency of lagging chromosomes in USP44^−/−^ mouse embryonic fibroblast (MEF) cells [53]. An increase in the numbers of lagging chromosomes mainly occurs when a single kinetochore is attached to both spindle poles, which is called merotelic attachment [54]. Several mitotic defects have been reported that promote the formation of merotelic attachments, including incomplete separation of centrosomes prior to NEBD [55]. In USP44^−/−^ MEF cells, abnormal spindle geometry and incomplete centrosome separation are increased, indicating that defects in these processes due to USP44 loss of function result in mitotic chromosome missegregation and aneuploidy. USP44 directly interacts with the centrosome component centrin and localizes to the centrosome during interphase. The DUB activity of USP44 and its ability to interact with centrin are critical for preventing chromosome lagging. Aneuploidy is very common in human lung adenocarcinoma, and reduced expression of USP44 is frequently observed in patients with lung adenocarcinoma [53]. In other words, reduced USP44 levels increase aneuploidy, which is associated with aggressive tumorigenesis.

### 3.2. USP9X

USP9X has been implicated in several disorders, including X-linked intellectual disability [56], Parkinson’s disease [57] and various types of malignancies [58,59,60]. In particular, USP9X functions related to mitosis are known to be associated with cancer development and progression. During mitosis, USP9X regulates chromosome alignment and segregation by regulating proper targeting of survivin and Aurora B to the centromeres and association of survivin with the centromeres [61]. Aurora B constitutes the catalytic subunit of the chromosomal passenger complex (CPC), which contains three regulatory subunits, namely, survivin, INCENP, and borealin. The CPC localization dynamically changes from the centromeres to spindle mid-zone during the metaphase–anaphase transition, and such relocation is essential for the activities and functions of the CPC proteins [62]. They regulate key mitotic events that are involved in chromosome condensation, correction of erroneous kinetochore–microtubule attachments, activation of the SAC, and cytokinesis. Therefore, the CPC is required for a successful cell division [63]. The depletion of USP9X leads to the accumulation of survivin and Aurora B on misaligned chromosomes, resulting in the induction of mitotic centromere-associated kinesin (MCAK) phosphorylation by Aurora B at the kinetochore. MCAK is a microtubule depolymerase that is critical for kinetochore–microtubule attachment [64]. Thus, MCAK phosphorylation by Aurora B may prevent its microtubule depolymerizing activity while correcting chromosome misalignments [61].

USP9X has previously been reported to deubiquitinate and consequently stabilize the prosurvival BCL2 family member MCL1 by preventing its proteasomal degradation, thereby promoting cell survival and contributing to chemoresistance in B-cell lymphoma [58]. However, independent of MCL1 status, USP9X promotes mitotic survival and resistance to spindle poisons by stabilizing the inhibitor of apoptosis protein (IAP) family member XIAP under the conditions of SAC-induced mitotic arrest [65]. Indeed, human aggressive B-cell lymphoma displays high USP9X-XIAP expression levels, which increase chemoresistance [65]. Hence, USP9X is a potential prognostic and therapeutic target in aggressive B-cell lymphoma. In addition, USP9X targets CEP131, a centrosome-associated CDC42 effector protein (CEP) family protein [66]. CEP131 is required for proper centrosome duplication and cilia formation before mitosis begins, suggesting that CEP131 might be associated with cancer development and progression [67]. USP9X is co-localized with CEP131 in centrosome and promotes CEP131 stabilization by deubiquitination. USP9X-mediated deubiquitination and stabilization of CEP131 lead to centrosome over-duplication (amplification), resulting in chromosome instability and mitotic aberrations [66].

Recently, Skowyra et al. showed that USP9X inhibits the degradation of SAC-controlled APC/C substrates, such as cyclin B1, cyclin A, and NIMA-related kinase 2A (NEK2A) during a mitotic arrest (Figure 2b). Specifically, USP9X restricts APC/C-mediated MCC turnover, thereby strengthening the SAC to protect from chromosomal instability (CIN). These USP9X functions are similar to those of USP44, implying that USP9X might be a potential therapeutic target in the treatment of various cancers [68].

### 3.3. Cylindromatosis (CYLD)

CYLD is a deubiquitinating enzyme that cleaves Lys63-linked polyubiquitin chains off its target proteins [69]. Originally identified as a tumor suppressor gene, CYLD is now known to be involved in the regulation of cell proliferation and is mutated in multiple tumors of skin appendages, referred to as cylindromas [70]. Additionally, a tumor suppressor function of CYLD has been described in several other malignancies, such as melanoma [71], salivary gland cancer [72,73,74], cervical cancer [75], hepatocellular carcinoma [76,77], and lung cancer [78]. This DUB has been extensively studied in the context of NF-κB signaling [79]. Loss of CYLD function causes failure to remove the Lys63-linked polyubiquitin chains on the proteins upstream of NF-κB, including TNF receptor-associated factor 2/6 (TRAF2/6), receptor-interacting serine/threonine-protein kinase 1/2 (RIPK1/2), and tumor necrosis factor receptor 1 (TNFR1), resulting in misactivation of NF-κB signaling and consequently promoting cell transformation while inhibiting apoptosis [80,81,82,83,84]. Furthermore, CYLD has been reported as an essential mediator of necrosis, which is a caspase-independent programmed cell death induced by tumor necrosis factor-α (TNF-α) or several triglyceride-rich lipoproteins (TRLs) [85,86,87]. In addition to cell survival, CYLD controls multiple cellular processes such as cell proliferation and inflammation by regulating the c-Jun N-terminal kinase (JNK) [80,81,82], Wnt [88], p38 mitogen-activated protein kinase (MAPK) [89], and protein kinase B (Akt) signaling pathways [90]. In cell cycle progression, CYLD regulates entry into mitosis independent of its canonical role in the regulation of the NF-κB pathway [70]. CYLD protein levels are regulated throughout the cell cycle. Particularly, the protein levels rapidly decrease as cells exit from mitosis. Moreover, CYLD localizes to microtubules during interphase and migrates to the midbody during telophase, thus having an important role in the regulation of cell-cycle. Stegmeier et al. observed impaired CDC25 in CYLD-depleted cells alongside delayed entry into mitosis. In contrast, overexpression of CYLD leads to an increase in the number of cells with fragmented or multiple nuclei, reflecting impairment in chromosome segregation and cytokinesis. Interestingly, PLK1 has been identified as a potential substrate of CYLD using a proteomic approach, suggesting that CYLD and PLK1 might together regulate mitotic entry and cytokinesis [70] (Figure 2c). CYLD also regulates mitotic spindle orientation through its dual function as deubiquitinating the cell polarity protein Disheveled (Dvl) and stabilizing the astral microtubules [91]. CYLD-mediated deubiquitination of Dvl stimulates the formation of the Dvl-NuMA-dynein/dynactin complex at the cell cortex causes the astral microtubules to be pulled, which rotates the spindle [91,92] (Figure 2a). Meanwhile, two amino-terminal cytoskeleton-associated protein glycine-rich (CAP-Gly) domains of CYLD interact with the astral microtubules and increase their stability [91]. With these two functions, CYLD promotes proper spindle orientation and eventually contributes to the control of cell division. Recently, another function of CYLD during mitosis has been revealed. CYLD interacts with a centrosome protein, CEP192, which plays a critical role in centrosome maturation and has a more specific role in the organization of the mitotic microtubules [93,94]. CYLD depletion mitigates the spindle assembly defects observed in CEP192-depleted cells even though lack of CYLD alone has no effect on the spindle assembly. Thus, CYLD might inhibit bipolar spindle assembly at least in the absence of CEP192 [93].

### 3.4. USP35 and Cezanne

USP35 functions as a mitotic regulator by deubiquitinating Aurora B kinase and maintaining its stability during mitosis [95]. Aurora B is an important kinase involved in dynamic cellular events in mitosis, including the regulation of kinetochore–microtubule dynamics, activation of the SAC, and completion of cytokinesis [63]. The stability and localization of Aurora B are important for its functions, and these two features of Aurora B are regulated by two E3 ubiquitin ligases, APC/C and CRL3-based complex, respectively. At the end of mitosis, APC/C is activated by co-activator CDH1 and conjugates Lys11-linked polyubiquitin chains to Aurora B, which is consequently degraded, thereby permitting cells to exit from mitosis into the G1 phase [96,97]. The CRL3-based complex regulates the dynamic behavior of Aurora B, which is located at the centromeres during early mitosis but migrates from the centromere to spindle mid-zone during the metaphase–anaphase transition. CRL3-mediated ubiquitination of Aurora B promotes this relocation, resulting in the completion of cytokinesis [35,36]. USP35-depleted cells display severe mitotic defects, such as chromosome misalignment, lagging chromosomes, multipolar spindles, and cytokinesis failure, generating daughter cells that exhibit genetic instability and aneuploidy, and these errors consequently cause tumorigenesis [95]. However, the defects observed in USP35-depleted cells are rescued by enforced expression of Aurora B. These results suggest that the effect of USP35 on the regulation of mitosis is mediated in an Aurora B-dependent manner. USP35 regulates the Aurora B protein levels during mitosis without affecting the localization of Aurora B. In other words, USP35 inhibits APC/C-CDH1-induced ubiquitination of Aurora B by cleaving Lys11-linked polyubiquitin chains and prevents it from proteasomal degradation (Figure 2d). Moreover, USP35-induced deubiquitination of Aurora B affects its activity. USP35 increases the phosphorylation of histone H3, indicating that USP35 is involved in the downstream signaling of Aurora B. In conclusion, USP35 is a DUB essential for the faithful progression of mitosis by maintaining the stability and function of Aurora B [95].

While USP35 counteracts the ubiquitination effect of APC/C on Aurora B, Cezanne antagonizes the degradation of other APC/C substrates without the involvement of Aurora B. Cezanne, which is also called OTUD7B, belongs to the OTU family that has ubiquitin linkage specificity [98], and this DUB can specifically dissociate Lys11-linked ubiquitin chains [98,99,100]. During mitosis, Lys11-linked ubiquitin chains are formed by APC/C [101,102,103]. Cezanne is a cell cycle-regulated DUB whose expression coincides with the timing of APC/C activation. Additionally, Cezanne binds to and rapidly deubiquitinates APC/C substrates, such as forkhead box protein M1 (FoxM1), Aurora A, and cyclin B1 by excising Lys11-linked ubiquitin chains, thereby antagonizing the degradation of APC/C substrates during mitosis (Figure 2d). However, degradation of Aurora B is unaffected by Cezanne. Similar to USP35, the depletion of Cezanne also significantly increases the frequency of lagging of misaligned chromosomes and micronuclei formation, leading to aneuploidy. Therefore, Cezanne regulates proper mitotic progression and exit by counteracting the activity of APC/C [104].

## 4. Conclusions

As reviewed here, ubiquitination and deubiquitination catalyzed by E3 ubiquitin ligases and DUBs, respectively, regulate mitotic progression at all stages of mitosis. Ubiquitin-dependent modifications during mitosis have been shown to play important roles in the regulation of the key components of several events, including chromosome condensation, alignment, and segregation, for a faithful mitotic progression. Additionally, the misregulated expression of DUBs involved in the modifications supports aberrant mitotic progression, leading to the development of many types of cancers (Table 1). As shown, abundant evidence from multiple reports suggests that DUBs can act as oncogenes or tumor suppressor genes by controlling mitosis, and thus they can serve as potential drug targets in cancer therapeutics. Therefore, identification of new DUBs associated with mitosis will contribute to the development of promising chemotherapeutic agents for various cancers.

## Figures and Tables

**Table 1:** Overview of the involvement of DUBs in mitosis.

DUB	Type	Substrate(s)	Function(s)	Cancer(s)	References
USP44	USP	CDC20	Regulation of the SAC	Lung adenocarcinoma	[43,53]
USP9X	USP	Survivin, XIAP, CEP131, APC/C substrates	Regulation of CPC functions, centrosome duplication, SAC	Lymphoma, myeloma, ductal, colon, prostate, and small-cell lung adenocarcinomas, glioblastoma, medulloblastoma	[60,61,65,66,68,105,106]
CYLD	USP	PLK1, Dvl, CEP192	Regulation of mitotic entry, cytokinesis, spindle orientation, and assembly	Cylindromas, melanoma, salivary gland, cervical, and lung cancers, hepatocellular carcinoma	[68,69,70,71,72,73,74,75,76,90,91,93]
USP35	USP	Aurora B	Regulation of chromosome alignment and segregation, and cytokinesis	Breast and lung cancers	[95,107]
Cezanne	OTU	APC/C substrates	Counteracting APC/C activity	Breast cancer	[98]
USP4	USP		Regulation of the SAC	Lung and breast cancers	[108,109]
BRISC	JAMM	NuMA	Regulation of bipolar spindle assembly	Breast cancer	[41]
USP11	USP	RAE1	Regulation of bipolar spindle assembly	Breast and pancreatic cancers	[42,110,111]
USP39	USP	Splicing of Aurora B mRNA	Regulation of the SAC	Ovarian cancer, glioma	[112,113,114]
Ubp-M (USP16)	USP	H2A, PLK1	Regulation of chromosome alignment and segregation, DNA damage response	Hepatocellular carcinoma	[115,116,117]
USP7	USP	Claspin	Counteracting SCF^βTrCP^-mediated claspin degradation, DNA damage response	Myeloma, prostate cancer, neuroblastoma, gliomas	[118,119,120,121]
USP28	USP	P53	P53-dependent cell cycle arrest in response to delayed mitosis		[122,123,124,125]
AMSH	JAMM	VAMP8	Regulation of cytokinesis		[126]

Listed are the names (DUB), subfamilies (Type), the known mitotic targets (Substrates), their general effect on the mitotic progression (Functions), DUB-related cancer types (Cancers), as well as important citations (References).
